# Therapeutic efficacy of molecular-targeted drugs for enthesitis in patients with PsA: a network meta-analysis

**DOI:** 10.1093/rap/rkaf077

**Published:** 2025-07-07

**Authors:** Daisuke Nakatsubo, Takayoshi Morita, Atsushi Kumanogoh

**Affiliations:** Department of Respiratory Medicine and Clinical Immunology, Graduate School of Medicine, Osaka University, Osaka, Japan; Department of Respiratory Medicine and Clinical Immunology, Graduate School of Medicine, Osaka University, Osaka, Japan; The Centre for Rheumatic Diseases, Nara Medical University, Nara, Japan; Department of Respiratory Medicine and Clinical Immunology, Graduate School of Medicine, Osaka University, Osaka, Japan; Integrated Frontier Research for Medical Science Division, Institute for Open and Transdisciplinary Research Initiatives (OTRI), Osaka University, Suita, Japan; Centre for Infectious Diseases for Education and Research (CiDER), Osaka University, Suita, Japan

**Keywords:** PsA, enthesitis, treatment, DMARDs, network meta-analysis, Leeds enthesitis index (LEI)

## Abstract

**Objectives:**

Enthesitis plays a key role in the pathogenesis of PsA. This study aimed to evaluate the efficacy of molecular-targeted therapies for enthesitis in patients with PsA.

**Methods:**

In this network meta-analysis (PROSPERO registration number: CRD42024590257), studies published up to April 2025 were searched in PubMed, Web of Science, Scopus and ClinicalTrials.gov. Only randomized controlled trials assessing molecular-targeted therapies for enthesitis in PsA were included. The primary outcomes were enthesitis resolution rates and Leeds enthesitis index score reductions at 12 and 24 weeks. A random-effects model was employed to calculate risk differences (RDs) or standardized mean differences (SMDs) with 95% confidence intervals (CIs), analysing both drug classes and individual agents.

**Results:**

Out of 5762 screened studies, 28 randomized controlled trials were included, encompassing 10 383 patients with PsA presenting with enthesitis. Therapies included TNF–alpha inhibitors, Janus kinase inhibitors, IL-17/IL-17 receptor inhibitors, IL-23 inhibitors and a cytotoxic T lymphocyte–associated antigen-4 immunoglobulin. At 24 weeks, upadacitinib significantly improved the enthesitis resolution rate [RD: 11.24 (95% CI: 4.26 to 18.23)] and reduced the Leeds enthesitis index scores [SMD: −0.72 (95% CI: −1.36 to −0.09)] compared with adalimumab; meanwhile, other Janus kinase inhibitors did not. Certolizumab also reduced the Leeds enthesitis index scores [SMD: −0.92 (95% CI: −1.72 to −0.13)].

**Conclusion:**

This network meta-analysis identified the more therapeutic efficacy of upadacitinib and certolizumab for enthesitis in patients with PsA than those of other molecular-targeted drugs. Notably, efficacy varied among molecular-targeted therapies, even within drug classes, underscoring the need for tailored therapeutic strategies.

Key messages

- Upadacitinib and certolizumab significantly reduced LEI scores in patients with PsA compared with adalimumab.
- Only upadacitinib showed a higher remission rate of enthesitis in patients with PsA than adalimumab.
- Agents within the same drug class have varying therapeutic responses for enthesitis, suggesting drug-specific efficacy.

## Introduction

PsA, a chronic inflammatory disease associated with psoriasis, affects 0.10–0.25% of the general population and 15.5–23.8% of patients with psoriasis [1–3]. PsA occurs equally in both sexes, presenting with musculoskeletal symptoms, including peripheral arthritis, dactylitis, enthesitis and axial involvement [4]. Skin symptoms generally precede musculoskeletal symptoms, although musculoskeletal symptoms appear first in 10–15% of patients [5]. Enthesitis is a key mechanism driving PsA’s musculoskeletal manifestations [3], commonly detected in the Achilles tendon and spine via ultrasound and magnetic resonance imaging [6]. Poorly controlled, enthesitis can cause joint destruction and impair quality of life [7].

Biomechanical stress and immune responses are important in enthesitis pathogenesis [6]. Entheses, where tendons and ligaments attach to the bone, endure repetitive mechanical stress, leading to transcortical microvessel vasodilation [6], consequently triggering innate immune cells (neutrophils and macrophages) infiltrate [6, 8]. Inflammation can also extend into the synovium via the synovio–entheseal complex, potentially causing arthritis [9]. Bone marrow–derived myeloid cells at the entheses produce IL-23, which activates innate immune cells to release IL-17A, thereby maintaining or even amplifying the inflammation [10, 11]. Chronic enthesitis leads to new bone formation and erosion at tendon attachment sites, driving PsA-specific pathology [6]. Therefore, targeting enthesitis directly in PsA management is needed rather than focusing solely on arthritis control.

The IL-23/IL-17 axis’ importance is reinforced by genetic and immunological research [12–14]. Animal model studies have further confirmed the role of this axis in enthesitis pathogenesis [15, 16]. Activated innate immune cells at the entheses produce IL-23 and IL-12, stimulating T helper (Th) 1 and Th17 cells, with Th17 cells releasing IL-17, which amplifies inflammation [10, 17]. Such inflammation is exacerbated by TNF–alpha (TNF-α) secreted by myeloid cells, including macrophages [17]. These proinflammatory cytokines drive enthesitis onset and progression, making the IL-23/IL-17 axis and TNF-α key therapeutic targets [18].

Conventional PsA treatments include NSAIDs, glucocorticoids, methotrexate, sulfasalazine, leflunomide and ciclosporin. However, considering our deepened understanding of PsA’s immune-mediated mechanisms, particularly those driven by the IL-23/IL-17 axis, immunologically targeted biological DMARDs (bDMARDs) and targeted synthetic DMARDs (tsDMARDs) have become standard treatments. Although they are included in international guidelines from the Group for Research and Assessment of Psoriasis and Psoriatic Arthritis [19], the European Alliance of Associations for Rheumatology [20] and the American College of Rheumatology (ACR) [5], their efficacy for enthesitis in PsA remains insufficiently evaluated.

This study aimed to evaluate the efficacy of molecular-targeted therapies in patients with PsA presenting with enthesitis in comparison with placebo or TNF-α inhibitors (TNFis), especially adalimumab, and identify the latest evidence on their efficacy for PsA-related enthesitis. Using a NMA, this research assessed enthesitis resolution rates and Leeds enthesitis index (LEI) score reductions to inform optimal therapeutic strategies.

## Methods

### Registration

An NMA was conducted to assess the efficacy of molecular-targeted therapies for enthesitis in patients with PsA. It was approved by the University of Osaka Hospital Ethical Review Board (approval no.: 24219), conforming to the Cochrane Handbook for Systematic Reviews of Interventions (version 6.5) [21] and the Preferred Reporting Items for Systematic Reviews and Meta-Analyses (PRISMA) statement [22] for reporting, including the PRISMA-NMA extension. The protocol was prospectively registered in PROSPERO (registration number: CRD42024590257). Supplementary Table S1, available at *Rheumatology Advances in Practice* online shows the PRISMA-NMA extension checklist.

### Search strategy

Databases such as PubMed, Web of Science, Scopus and ClinicalTrials.gov were systematically searched using the following search query: (‘Psoriatic Arthritis’ OR ‘Psoriatic disease’ OR ‘Spondyloarthropathy’ OR ‘Spondyloarthritis’) AND ‘Enthesitis’ AND (‘Treatment’ OR ‘Therapy’) (Supplementary Table S2, available at *Rheumatology Advances in Practice* online). We included studies published between January 1986 and April 2025 with no language restrictions, completing the final search on 30 April 2025.

### Selection criteria

Inclusion criteria for the screened studies were as follows: reported original human research focusing on patients with PsA and enthesitis treated with molecular-targeted therapies; included relevant terms in the title or abstract; reported PsA diagnosis via the ‘classification criteria for psoriatic arthritis’ [23]; and were available in full text online. Conversely, we excluded studies that involved paediatric patients (<18 years old), lacked data on LEI score reductions or LEI = 0 achievement rates at 12 or 24 weeks, were case reports and were duplicate studies across databases.

### Data extraction

Extracted data included author names, publication year, follow-up period, sample size, mean age, female patient percentage, disease duration, proportion of patients with enthesitis, baseline LEI scores, prior molecular-targeted therapy use and intervention therapy details. Outcomes included LEI score changes, LEI = 0 achievement rates at 12 or 24 weeks and their standard errors. Additionally, ACR70 response rates between 12 and 24 weeks (with standard errors) were extracted.

### Quality assessment

Two authors (D.N. and T.M.) independently screened studies, resolving disagreements through discussion. The National Heart, Lung, and Blood Institute’s (NHLBI’s) quality assessment tool for observational cohort and cross-sectional studies was used for assessing study quality [24]. Publication bias was evaluated using a comparison-adjusted funnel plot, as recommended by the Cochrane Collaboration [21].

### Data synthesis and statistical analysis

Data were statistically analysed using R version 4.5.0 (R Project for Statistical Computing) [25] and EZR^®^ version 1.68 [26]. Patients receiving molecular-targeted therapies formed the intervention group, categorized by drug class, i.e., drugs with similar mechanisms of action. The placebo group included patients treated with conventional synthetic DMARDs, NSAIDs or oral corticosteroids, excluding molecular-targeted therapies.

Standardized mean differences (SMDs) or risk differences (RDs), with their 95% confidence intervals (CIs), were used to compare LEI score changes and LEI = 0 achievement rates, respectively, between the intervention group and either the placebo group or the TNFis group. Direct and indirect evidence and pool effect sizes were integrated using a random-effects model. Pooled results were reported with SMDs or RDs and their 95% CIs and visualized using forest plots. A *P*-value below 0.05 was considered statistically significant. Heterogeneity was assessed using the I^2^ statistic, employing thresholds of 25% (low), 50% (moderate) and 75% (high) [27].

### Additional analyses

In addition to drug class–based analysis, subgroup analysis was conducted to compare individual molecular-targeted therapies with placebos or adalimumab.

### Role of the funding source

The study funders had no role in the study design, data collection, data analysis, data interpretation and manuscript writing.

## Results

### Study selection

**Figure 1.**
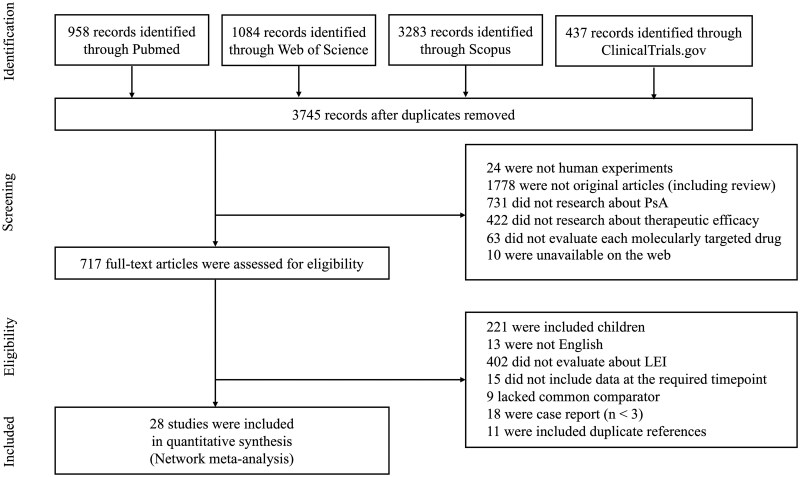
Flow diagram of study selection

Overall, 3745 unique studies were retrieved from PubMed (958), Web of Science (1084), Scopus (3283) and ClinicalTrials.gov (437). Among these, 717 met the inclusion criteria; 689 studied were excluded. Ultimately, 28 studies were included in the NMA (Fig. 1) [28–55]. All included studies were randomized controlled trials (RCTs).

### Study characteristics and patient backgrounds

Table 1 and Supplementary Table S3, available at *Rheumatology Advances in Practice* online summarize the 28 studies included in the NMA. The evaluated molecular-targeted therapies included TNFis (adalimumab, 7 studies; certolizumab, 1); Janus kinase inhibitors (JAKis: filgotinib, 1; tofacitinib, 3; upadacitinib, 3); IL-17/IL-17 receptor inhibitors (IL-17is/IL-17Ris: bimekizumab, 2; brodalumab, 3; ixekizumab, 5; secukinumab, 3); IL-23 inhibitors (IL-23is: guselkumab, 4; risankizumab, 2; tildrakizumab, 1) and a cytotoxic T lymphocyte–associated antigen-4 immunoglobulin (CTLA-4Ig: abatacept, 1). The ECLIPSA study was included in class-level comparisons only because dose-specific data for ustekinumab and individual TNFi agents were unavailable [55]. Seventeen studies examined different dosages or dosing intervals of the same drug. Seven studies on bimekizumab, ixekizumab, secukinumab, tofacitinib and upadacitinib used adalimumab as an active control.

**Table 1.** Study characteristics included in the NMA

Author	Registered number	Publication year	Drug class	Treatment	Patient number	Outcome			
						LEI = 0 (12 weeks)	LEI = 0 (24 weeks)	ΔLEI (12 weeks)	ΔLEI (24 weeks)
Mease *et al.* [28]	NCT01860976	2017	CTLA-4Ig	Abatacept 125 mg	140	―	◯	―	―
				Placebo	132	―	◯	―	―
Mease *et al.* [29]	BE OPTIMAL (NCT03895203)	2024	IL-17i	Bimekizumab 160 mg Q4W	143	◯	◯	―	―
			TNFi	Adalimumab 40 mg	36	◯	◯	―	―
				placebo	70	◯	―	―	―
	BE COMPLETE (NCT03896581)		IL-17i	Bimekizumab 160 mg Q4W	106	◯	―	―	―
				placebo	36	◯	―	―	―
Mease *et al.* [30]	NCT01516957	2014	IL-17Ri	Brodalumab 140 mg	41	―	―	◯	―
			IL-17Ri	Brodalumab 280 mg	32	―	―	◯	―
				placebo	45	―	―	◯	―
Mease *et al.* [31]	AMVISION-1 (NCT02029495)	2021	IL-17Ri	Brodalumab 140 mg	107	―	◯	―	―
			IL-17Ri	Brodalumab 210 mg	93	―	◯	―	―
				placebo	93	―	◯	―	―
	AMVISION-2 (NCT02024646)		IL-17Ri	Brodalumab 140 mg	101	―	◯	―	―
			IL-17Ri	Brodalumab 210 mg	92	―	◯	―	―
				placebo	100	―	◯	―	―
Mease *et al.* [32]	RAPID-PsA (NCT01087788)	2014	TNFi	Certolizumab pegol 200 mg Q2W	88	―	―	―	◯
			TNFi	Certolizumab pegol 400 mg Q4W	84	―	―	―	◯
				placebo	91	―	―	―	◯
Mease *et al.* [33]	EQUATO (NCT03101670)	2018	JAKi	Filgotinib 200 mg	38	◯	―	◯	―
				placebo	49	◯	―	◯	―
Mease *et al.* [34]	NCT02319759	2020	IL-23i	Guselkumab 100 mg Q8W	76	―	◯	―	―
				placebo	31	―	◯	―	―
Deodhar *et al.* [35]	DISCOVER-1 (NCT03162796)	2020	IL-23i	Guselkumab 100 mg Q4W	73	―	◯	―	―
			IL-23i	Guselkumab 100 mg Q8W	72	―	◯	―	―
				placebo	77	―	◯	―	―
McInnes *et al.* [36]	DISCOVER-2 (NCT03158285)	2022	IL-23i	Guselkumab 100 mg Q4W	170	―	◯	―	◯
			IL-23i	Guselkumab 100 mg Q8W	158	―	◯	―	◯
				placebo	178	―	◯	―	◯
McGonagle *et al.* [37]	DISCOVER-1 (NCT03162796) and	2021	IL-23i	Guselkumab 100 mg Q4W	243	―	◯	―	◯
	DISCOVER-2 (NCT03158285)		IL-23i	Guselkumab 100 mg Q8W	230	―	◯	―	◯
				placebo	255	―	◯	―	◯
Mease *et al.* [38]	SPIRIT-P1 (NCT01695239)	2017	IL-17i	Ixekizumab 80 mg Q4W	70	◯	◯	◯	◯
			IL-17i	Ixekizumab 80 mg Q2W	59	◯	◯	◯	◯
			TNFi	Adalimumab 40 mg	56	◯	◯	◯	◯
				placebo	57	◯	◯	◯	◯
Nash *et al.* [39]	SPIRIT-P2 (NCT02349295)	2017	IL-17i	Ixekizumab 80 mg Q4W	68	◯	◯	◯	◯
			IL-17i	Ixekizumab 80 mg Q2W	84	◯	◯	◯	◯
				placebo	69	◯	◯	◯	◯
Gladman *et al.* [40]	SPIRIT-P1 (NCT01695239) and	2019	IL-17i	Ixekizumab 80 mg Q4W	136	―	◯	―	―
	SPIRIT-P2 (NCT02349295)		IL-17i	Ixekizumab 80 mg Q2W	141	―	◯	―	―
				placebo	126	―	◯	―	―
Kristensen *et al.* [41]	SPIRIT-H2H (Group A) (NCT03151551)	2022	IL-17i	Ixekizumab 80 mg Q4W	25	―	◯	―	―
			TNFi	Adalimumab 40 mg	28	―	◯	―	―
	SPIRIT-H2H (Group B) (NCT03151551)		IL-17i	Ixekizumab 80 mg Q4W	134	―	◯	―	―
			TNFi	Adalimumab 40 mg	118	―	◯	―	―
Östör *et al.* [42]	KEEPsAKE 2 (NCT03671148)	2022	IL-23i	Risankizumab 150 mg	224	―	◯	―	―
				placebo	219	―	◯	―	―
Kwatra *et al.* [43]	KEEPsAKE 1 (NCT03675308) and	2024	IL-23i	Risankizumab 150 mg	429	◯	◯	◯	◯
	KEEPsAKE 2 (NCT03671148)			placebo	426	◯	◯	◯	◯
Nash *et al.* [44]	FUTURE 3 (NCT01989468)	2018	IL-17i	Secukinumab 150 mg	95	―	◯	―	―
			IL-17i	Secukinumab 300 mg	88	―	◯	―	―
Mease *et al.* [45]	FUTURE 5 (NCT02404350)	2018	IL-17i	Secukinumab 150 mg	141	◯	◯	―	―
			IL-17i	Secukinumab 300 mg	140	◯	◯	―	―
				placebo	192	◯	◯	―	―
McInnes *et al.* [46]	EXCEED (NCT02745080)	2020	IL-17i	Secukinumab 300 mg	234	◯	―	―	―
			TNFi	Adalimumab 40 mg	264	◯	―	―	―
Kaeley *et al.* [47]	EXCEED (NCT02745080)	2024	IL-17i	Secukinumab 300 mg	234	―	◯	―	◯
			TNFi	Adalimumab 40 mg	264	―	◯	―	◯
Mease *et al.* [48]	NCT02980692	2021	IL-23i	Tildrakizumab 100 mg Q12W	51	―	―	―	◯
			IL-23i	Tildrakizumab 200 mg Q12W	43	―	―	―	◯
			IL-23i	Tildrakizumab 200 mg Q4W	48	―	―	―	◯
			IL-23i	Tildrakizumab 20 mg Q12W	55	―	―	―	◯
				placebo	43	―	―	―	◯
Mease *et al.* [49]	OPAL Broaden (NCT01877668)	2017	JAKi	Tofacitinib 5 mg	70	◯	―	◯	◯
			JAKi	Tofacitinib 10 mg	63	◯	―	◯	◯
			TNFi	Adalimumab 40 mg	73	◯	―	◯	◯
				placebo	63	◯	―	◯	―
Gladman *et al.* [50]	OPAL Beyond (NCT01882439)	2017	JAKi	Tofacitinib 5 mg	79	◯	―	◯	◯
			JAKi	Tofacitinib 10 mg	86	◯	―	◯	◯
				placebo	82	◯	―	◯	―
Nash *et al.* [51]	OPAL Broaden (NCT01877668) and	2018	JAKi	Tofacitinib 5 mg	158	◯	―	◯	◯
	OPAL Beyond (NCT01882439)		JAKi	Tofacitinib 10 mg	163	◯	―	◯	◯
				placebo	158	◯	―	◯	―
McInnes *et al.* [52]	SELECT-PsA 1 (NCT03104400)	2021	JAKi	Upadacitinib 15 mg	270	◯	◯	―	―
			JAKi	Upadacitinib 30 mg	267	◯	◯	―	―
			TNFi	Adalimumab 40 mg	265	◯	◯	―	―
				placebo	241	◯	◯	―	―
Mease *et al.* [53]	SELECT-PsA 2 (NCT03104374)	2021	JAKi	Upadacitinib 15 mg	133	◯	◯	―	―
			JAKi	Upadacitinib 30 mg	152	◯	◯	―	―
				placebo	144	◯	◯	―	―
Cantini *et al.* [54]	SELECT-PsA 1 (NCT03104400) and	2024	JAKi	Upadacitinib 15 mg	387	◯	◯	◯	◯
	SELECT-PsA 2 (NCT03104374)			placebo	353	◯	◯	◯	◯
Araujo *et al.* [55]	ECLIPSA (EudraCT 2017-003799-29)	2019	IL-23i	Ustekinumab (<100 kg body weight: 45 mg; >100 kg body weight; 90 mg)	23	―	◯	◯	◯
			TNFi	Adalimumab: *n *= 10, Certolizumab: *n *= 6, Etanercept: *n *= 5, Infliximab: *n *= 3	24	―	◯	◯	◯

CTLA-4Ig: cytotoxic T lymphocyte–associated antigen-4 immunoglobulin; IL-17i: interleukin-17 inhibitor; IL-17Ri: interleukin-17 receptor inhibitor; IL-23i: interleukin-23 inhibitor; JAKi: Janus kinase inhibitor; LEI: Leeds enthesitis index; NMA: network meta-analysis; Q2W: once every 2 weeks; Q4W: once every 4 weeks; Q8W: once every 8 weeks; Q12W: once every 12 weeks; TNFi: tumour necrosis factor–alpha inhibitor.

In total, 7565 patients belonged to the intervention or active control groups, whereas 3330 belonged to the placebo group. Among them, 7047 in the intervention group and 3336 in the placebo group presented with enthesitis. Patients’ mean age was 44.9 to 54.1 years, with 25.0–71.0% being female. Mean duration of PsA was 5.1 to 11.0 years. All patients in five studies had a history of prior molecular-targeted therapy use. Six studies included only treatment-naive patients. In the remaining studies, the proportion of prior users was 1.0–63.6%. Baseline methotrexate users accounted for 22.6–100%, with additional treatments including sulfasalazine, leflunomide, hydroxychloroquine, oral corticosteroids and NSAIDs. Baseline LEI scores among patients with enthesitis were 1.6 to 3.4.

Study quality, which was assessed using the NHLBI quality assessment tool, scored at 12 to 14 points (Supplementary Table S4, available at *Rheumatology Advances in Practice* online). Regarding publication bias, funnel plots indicated symmetrical study distribution (Supplementary Figs S1 and S2, available at *Rheumatology Advances in Practice* online).

### Efficacy of drug classes on enthesitis resolution in patients with PsA

**Figure 2.**
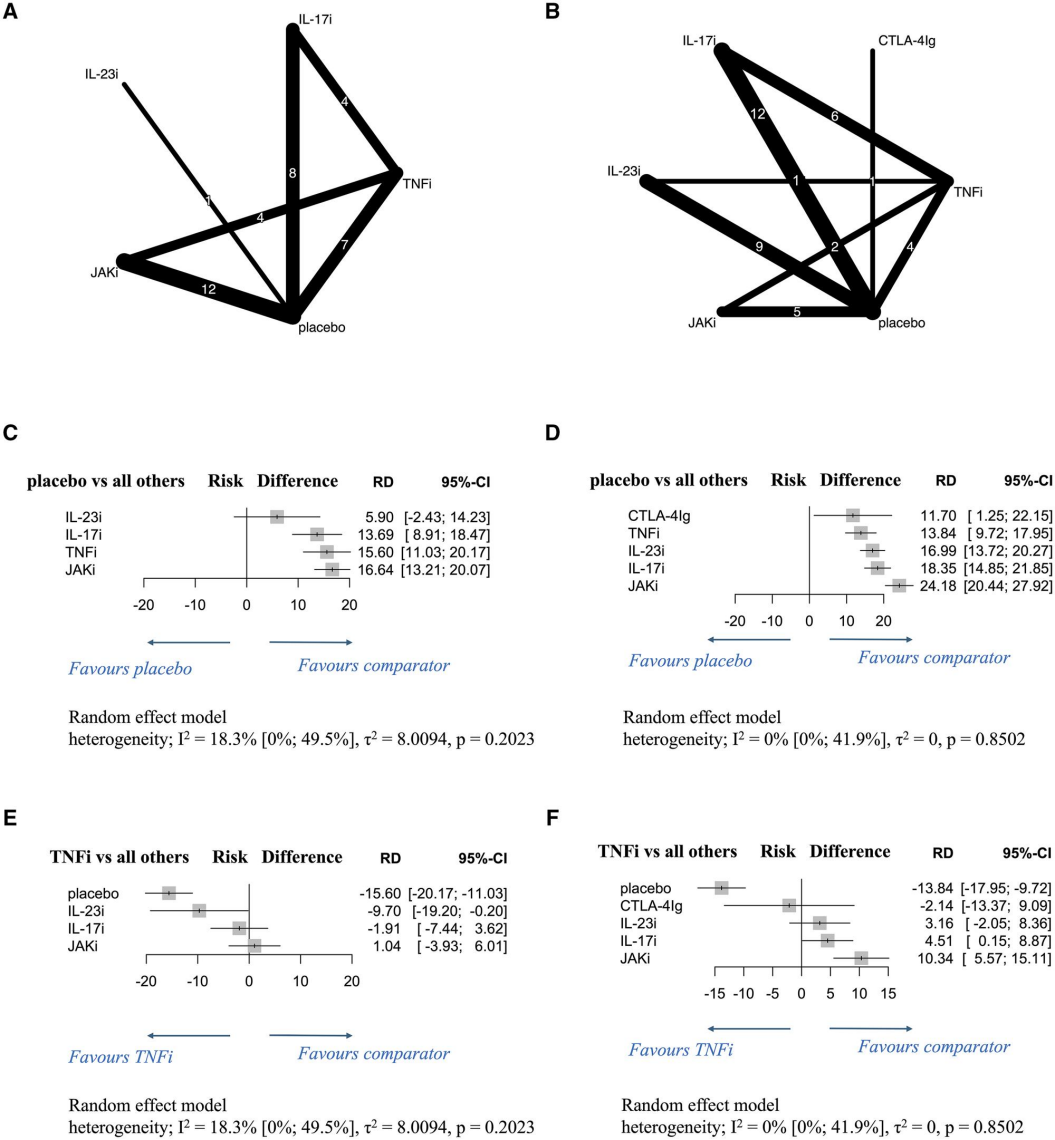
Network diagrams and forest plots comparing molecular-targeted drugs by drug classes for enthesitis resolution in patients with PsA. (**A, B**) Network diagrams show the enthesitis resolution rates at 12 weeks (**A**) and 24 weeks (**B**) of administering various molecular-targeted drugs to patients with PsA. The node size is proportional to the total number of patients randomized to each treatment; the edge line thickness is proportional to the total number of studies informing each comparison. (**C–F**) Forest plots present the enthesitis resolution rates at 12 weeks (**C, E**) and 24 weeks (**D, F**) in patients with PsA. Results are presented as risk differences with 95% confidence intervals, estimated using a random-effects model. Forest plots in **C** and **D** compare various molecular-targeted drugs with placebo, while those in **E** and **F** compare such drugs with TNFi. Heterogeneity across studies was assessed using the I^2^ statistic. Molecular-targeted drugs were compared by drug classes to assess therapeutic effect variations. CTLA-4Ig: cytotoxic T lymphocyte–associated antigen-4 immunoglobulin; IL-17i: interleukin-17 inhibitor; IL-17Ri: interleukin-17 receptor inhibitor; IL-23i: interleukin-23 inhibitor; JAKi: Janus kinase inhibitor; RD: risk difference; TNFi: tumour necrosis factor–alpha inhibitor; 95% CI: 95% confidence intervals

Fourteen studies assessed the efficacy of the following molecular-targeted therapies for enthesitis at 12 weeks of administration in the intervention group or the active control: TNFis: adalimumab 40 mg (adalimumab^40mg^) (5 studies); JAKIs: filgotinib 200 mg (filgotinib^200mg^) (1), tofacitinib 5 mg (tofacitinib^5mg^) (3) and 10 mg (tofacitinib^10mg^) (3), and upadacitinib 15 mg (upadacitinib^15mg^) (3) and 30 mg (upadacitinib^30mg^) (2); IL-17is: bimekizumab 160 mg (bimekizumab^160mg^) once every 4 weeks (Q4W) (2), ixekizumab 80 mg (ixekizumab^80mg^) once every 2 weeks (Q2W) (2) and Q4W (2), and secukinumab 150 mg (secukinumab^150mg^) (1) and 300 mg (secukinumab^300mg^) (2); IL-23is: risankizumab 150 mg (risankizumab^150mg^) (1) (Fig. 2A). Furthermore, 21 studies examined the efficacy of the following molecular-targeted therapies at 24 weeks of administration: TNFis: adalimumab^40mg^ (6 studies); JAKis: upadacitinib^15mg^ (3) and upadacitinib^30mg^ (2); IL-17is/IL-17Ris: bimekizumab^160mg^ Q4W (1), brodalumab 140 mg (brodalumab^140mg^) (2) and 210 mg (brodalumab^210mg^) (2), ixekizumab^80mg^ Q2W (3) and Q4W (5), and secukinumab^150mg^ (2) and secukinumab^300mg^ (3); IL-23is: guselkumab 100 mg (guselkumab^100mg^) Q4W (3) and once every 8 weeks (Q8W) (4) and risankizumab^150mg^ (2); CTLA-4Ig: abatacept 125 mg (abatacept^125mg^) (1) (Fig. 2B).

At 12 and 24 weeks, all therapies, except IL-23is at 12 weeks, significantly improved PsA-related enthesitis resolution rates compared with the placebo (Fig. 2C and D). At both time points, JAKis demonstrated highest resolution rates [12-week RD: 16.64 (95% CI: 13.21 to 20.07); 24-week RD: 24.18 (95% CI: 20.44 to 27.92)]. Low heterogeneity was observed at 12 weeks (I^2^ = 18.3%, *P *= 0.2023) and no heterogeneity at 24 weeks (I^2^ = 0%, *P *= 0.8502). Enthesitis resolution rates at 12 weeks did not significantly differ between any drug class and TNFis (Fig. 2E); however, those at 24 weeks were significantly higher with JAKis and IL-17is than with TNFis, with JAKis showing the greatest effect [RD: 10.34 (95% CI: 5.57 to 15.11); Fig. 2F].

### Efficacy of individual drugs on enthesitis resolution rates in patients with PsA

**Figure 3.**
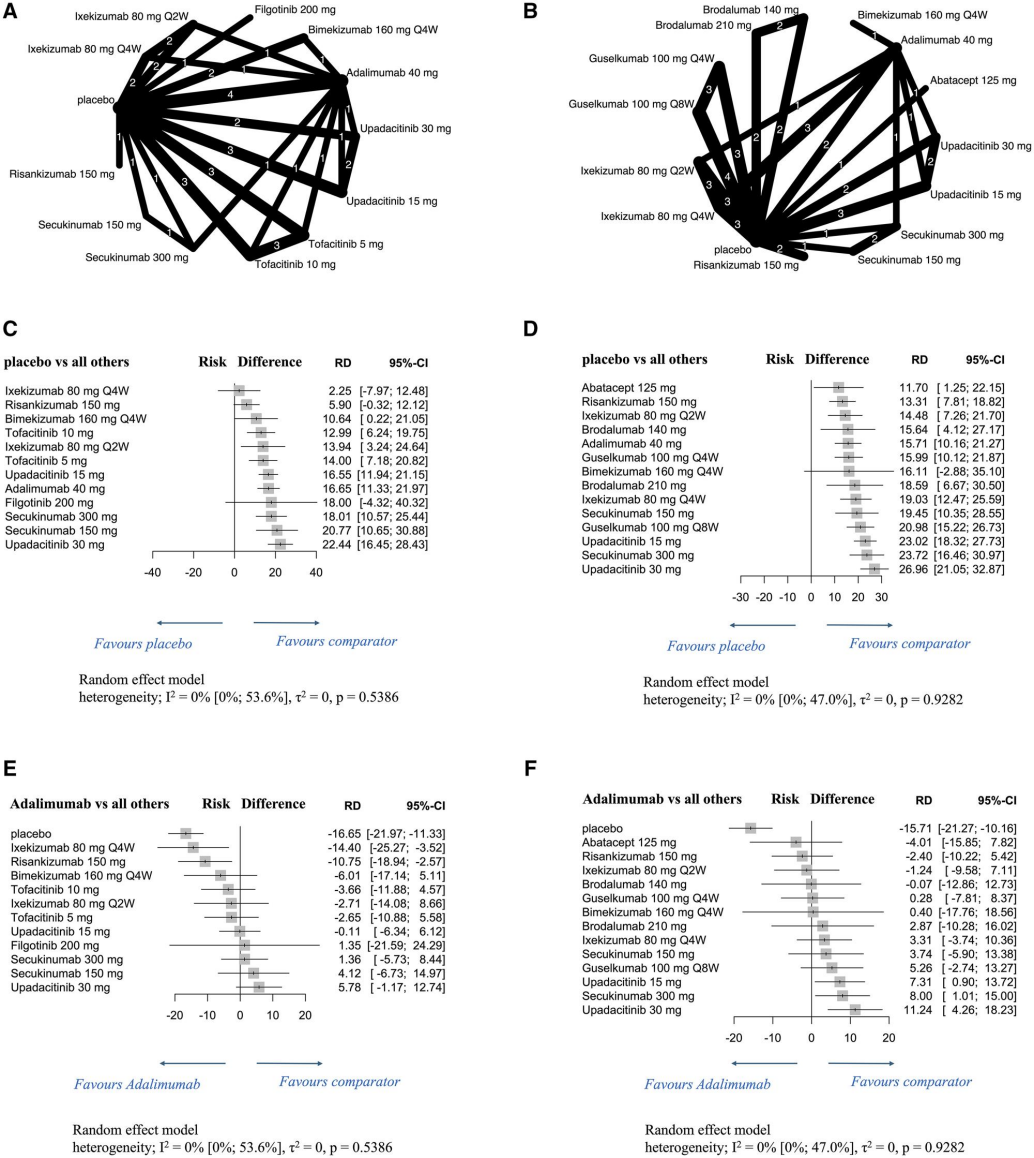
Network diagrams and forest plots evaluating individual molecular-targeted drugs for enthesitis resolution in patients with PsA. (**A, B**) Network diagrams show the enthesitis resolution rates at 12 weeks (**A**) and 24 weeks (**B**) of administering individual molecular-targeted drugs to patients with PsA. The node size is proportional to the total number of patients randomized to each treatment; the edge line thickness is proportional to the total number of studies informing each comparison. (**C–F**) Forest plots present the enthesitis resolution rates at 12 weeks (**C, E**) and 24 weeks (**D, F**) in patients with PsA. Results are presented as risk differences with 95% confidence intervals, estimated using a random-effects model. Forest plots in **C** and **D** compare individual molecular-targeted drugs with placebo, while those in **E** and **F** compare such drugs with adalimumab. Heterogeneity across studies was assessed using the I^2^ statistic. In this analysis, molecular-targeted drugs were evaluated individually. Q2W: once every 2 weeks; Q4W: once every 4 weeks; Q8W: once every 8 weeks; RD: risk difference; 95% CI: 95% confidence intervals

Network diagrams show enthesitis resolution rates of individual molecular-targeted drugs at 12 weeks (Fig. 3A) and 24 weeks (Fig. 3B) in patients with PsA. At 12 weeks, most therapies showed significantly higher enthesitis resolution rates than the placebo, with the highest rate observed for upadacitinib^30mg^ [RD: 22.44 (95% CI: 16.45 to 28.43)]. However, the rates did not significantly differ between the placebo and either ixekizumab^80mg^ Q4W, bimekizumab^160mg^ Q4W or filgotinib^200mg^ (Fig. 3C). At 24 weeks, all therapies significantly outperformed the placebo, except for bimekizumab^160mg^ Q4W, with upadacitinib^30mg^ demonstrating the highest resolution rate [RD: 26.96 (95% CI: 21.05 to 32.87); Fig. 3D]. No heterogeneity was observed at either time point (12 weeks: I^2^ = 0%, *P *= 0.5386; 24 weeks: I^2^ = 0%, *P *= 0.9282). At 12 weeks, no therapy exhibited a significantly higher resolution rate than that of adalimumab^40mg^ (Fig. 3E). However, at 24 weeks, upadacitinib^15mg^ and upadacitinib^30mg^ and secukinumab^300mg^ achieved significantly higher resolution rates than adalimumab^40mg^ [upadacitinib^15mg^ RD: 7.31 (95% CI: 0.90 to 13.72); upadacitinib^30mg^ RD: 11.24 (95% CI: 4.26 to 18.23); secukinumab^300mg^ RD: 8.00 (95% CI: 1.01 to 15.00); Fig. 3F].

### Efficacy of drug classes on LEI score reductions in patients with PsA

**Figure 4.**
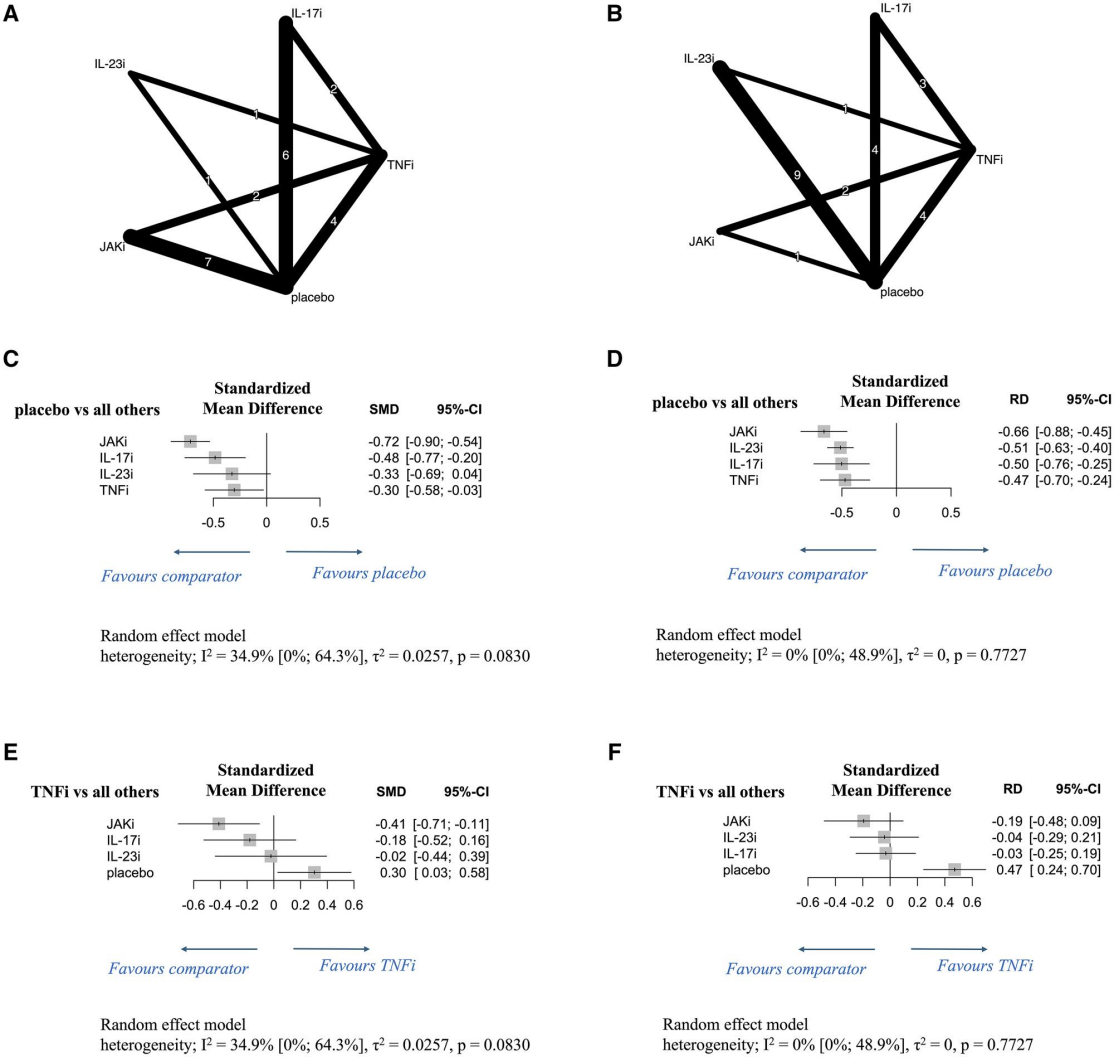
Network diagrams and forest plots comparing molecular-targeted drugs by drug classes for LEI score reduction in patients with PsA. (**A, B**) Network diagrams show LEI score reductions at 12 weeks (**A**) and 24 weeks (**B**) of administering various molecular-targeted drugs to patients with PsA. The node size is proportional to the total number of patients randomized to each treatment; the edge line thickness is proportional to the total number of studies informing each comparison. (**C–F**) Forest plots present LEI score reductions at 12 weeks (**C, E**) and 24 weeks (**D, F**) in patients with PsA. Results are presented as mean differences with 95% confidence intervals, estimated using a random-effects model. Forest plots in **C** and **D** compare various molecular-targeted drugs with placebo, while those in **E** and **F** compare them with TNFi. Heterogeneity across studies was assessed using the I^2^ statistic. Molecular-targeted drugs were compared by drug class to assess variations in therapeutic effects on LEI score reduction. IL-17i: interleukin-17 inhibitor; IL-17Ri: interleukin-17 receptor inhibitor; IL-23i: interleukin-23 inhibitor; JAKi: Janus kinase inhibitor; LEI: Leeds enthesitis index; SMD: standardized mean difference; TNFi: tumour necrosis factor–alpha inhibitor; 95% CI: 95% confidence intervals

Nine studies evaluated the following molecular-targeted therapies in the intervention group at 12 weeks: TNFis: adalimumab^40mg^ (2 studies); JAKis: filgotinib^200mg^ (1), tofacitinib^5mg^ (3) and tofacitinib^10mg^ (3), and upadacitinib^15mg^ (1); IL-17is/IL-17Ris: brodalumab^140mg^ (1) and brodalumab 280 mg (brodalumab^280mg^) (1) and ixekizumab^80mg^ Q2W (2) and Q4W (2); IL-23is: risankizumab^150mg^ (1) (Fig. 4A).

Twelve studies assessed the following molecular-targeted therapies at 24 weeks: TNFis: adalimumab^40mg^ (3), certolizumab 200 mg (certolizumab^200mg^) Q2W (1), and 400 mg (certolizumab^400mg^) Q4W (1); JAKis: tofacitinib^5mg^ (3) and tofacitinib^10mg^ (3) and upadacitinib^15mg^ (1); IL-17is/IL-17Ris: ixekizumab^80mg^ Q2W (2) and Q4W (2) and secukinumab^300mg^ (1); IL-23is: guselkumab^100mg^ Q4W (2) and Q8W (2), risankizumab^150mg^ (1), and tildrakizumab 200 mg Q4W (1) and once every 12 weeks (Q12W) (1), 100 mg Q12W (1) and 20 mg Q12W (1) (Fig. 4B).

At 12 weeks, IL-17is/IL-17Ris and JAKis significantly reduced LEI scores compared with the placebo [JAKis: SMD: −0.72 (95% CI: −0.90 to −0.54); IL-17is/IL-17Ris: SMD: −0.48 (95% CI: −0.77 to −0.20); Fig. 4C]; moderate heterogeneity was observed (I^2^ = 34.9%, *P *= 0.0830). At 24 weeks, all therapies significantly reduced LEI scores compared with the placebo, with the greatest reduction observed for JAKis [SMD: −0.66 (95% CI: −0.88 to −0.45); Fig. 4D], and no heterogeneity was observed (I^2^ = 0%, *P *= 0.7727). At 12 weeks, JAKis significantly outperformed TNFis in reducing LEI scores [SMD: −0.41 (95% CI: −0.71 to −0.11); Fig. 4E]. Conversely, no significant differences were observed among drug classes at 24 weeks (Fig. 4F).

### Efficacy of individual drugs on LEI score reductions in patients with PsA

**Figure 5.**
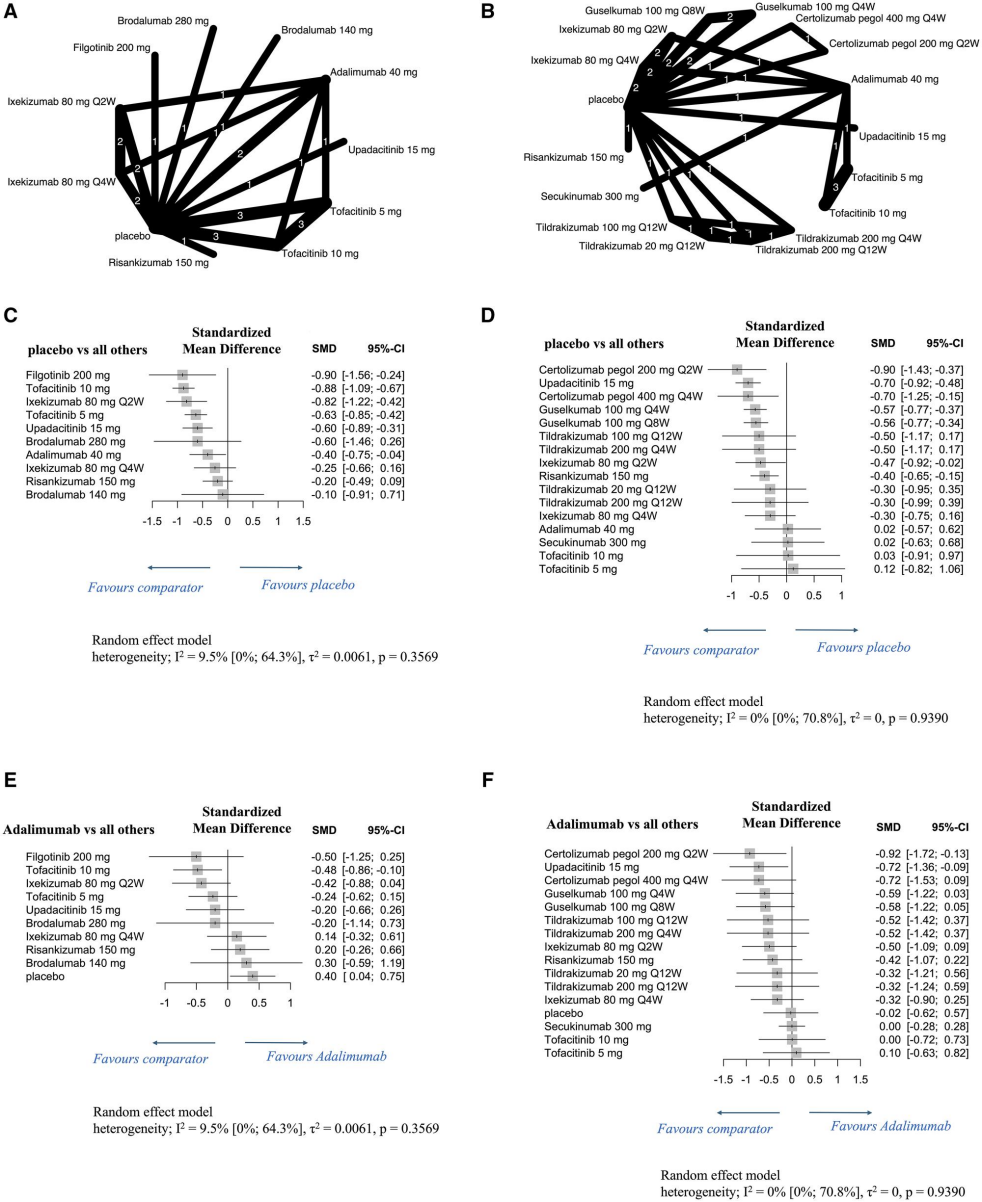
Network diagrams and forest plots evaluating individual molecular-targeted drugs for LEI score reduction in patients with PsA. (**A, B**) Network diagrams show LEI score reductions at 12 weeks (**A**) and 24 weeks (**B**) of administering individual molecular-targeted drugs to patients with PsA. The node size is proportional to the total number of patients randomized to each treatment; the edge line thickness is proportional to the total number of studies informing each comparison. (**C–F**) Forest plots present LEI score reductions at 12 weeks (**C, E**) and 24 weeks (**D, F**) in patients with PsA. Results are presented as mean differences with 95% confidence intervals, estimated using a random-effects model. Forest plots in **C** and **D** compare individual molecular-targeted drugs with placebo, while those in **E** and **F** compare such drugs with adalimumab. Heterogeneity across studies was assessed using the I^2^ statistic. The molecular-targeted drugs were evaluated individually to assess their specific effects on LEI score reduction. LEI: Leeds enthesitis index; SMD: standardized mean difference; Q2W: once every 2 weeks; Q4W: once every 4 weeks; Q8W: once every 8 weeks; Q12W: once every 12 weeks; 95% CI: 95% confidence intervals

Network diagrams show LEI score reductions of individual molecular-targeted drugs at 12 weeks (Fig. 5A) and 24 weeks (Fig. 5B) in patients with PsA.

At 12 weeks, filgotinib^200mg^, tofacitinib^5mg^ and tofacitinib^10mg^, upadacitinib^15mg^, ixekizumab^80mg^ Q2W and adalimumab^40mg^ significantly reduced the LEI scores compared with the placebo, with filgotinib^200mg^ [SMD: −0.90 (95% CI: −1.56 to −0.24)], tofacitinib^10mg^ [SMD: −0.88 (95% CI: −1.09 to −0.67)] and ixekizumab^80mg^ Q2W [SMD: −0.82 (95% CI: −1.22 to −0.42)] showing the most pronounced reductions (Fig. 5C). Low heterogeneity was observed (I^2^ = 9.5%, *P *= 0.3569). At 24 weeks, certolizumab^200mg^ Q2W, certolizumab^400mg^ Q4W, upadacitinib^15mg^, guselkumab^100mg^ Q4W and Q8W, ixekizumab^80mg^ Q2W and risankizumab^150mg^ significantly reduced the LEI scores compared with the placebo, with certolizumab^200mg^ Q2W [SMD: −0.90 (95% CI: −1.43 to −0.37)], certolizumab^400mg^ Q4W [SMD: −0.70 (95% CI: −1.25 to −0.15)] and upadacitinib^15mg^ [SMD: −0.70 (95% CI: −0.92 to −0.48)] demonstrating the greatest reductions (Fig. 5D). No heterogeneity was noted (I^2^ = 0%, *P *= 0.9390). At 12 weeks, significant LEI scores reductions were observed for tofacitinib^10mg^ [SMD: −0.48 (95% CI: −0.86 to −0.10)] (Fig. 5E). At 24 weeks, certolizumab^200mg^ Q2W [SMD: −0.92 (95% CI: −1.72 to −0.13)] and upadacitinib^15mg^ [SMD: −0.72 (95% CI: −1.36 to −0.09)] significantly outperformed adalimumab^40mg^ (Fig. 5F).

### Efficacy of drug classes and individual drugs on ACR70 response rates in patients with PsA

ACR70 response rates for all patients with PsA in the 21 included studies were assessed for 12 to 24 weeks (Supplementary Fig. S3A, available at *Rheumatology Advances in Practice* online). JAKis, TNFis, IL-17is/IL-17Ris and IL-23is demonstrated significantly higher rates of ACR70 response than the placebo (Supplementary Fig. S3B, available at *Rheumatology Advances in Practice* online). Moderate heterogeneity was observed (I^2^ = 63%, *P *< 0.0001). Notably, the ACR70 response rates did not significantly differ between any drug class and TNFis (Supplementary Fig. S3C, available at *Rheumatology Advances in Practice* online).

In individual drug analyses, upadacitinib^30mg^ achieved the highest ACR70 response rate compared with the placebo [RD: 27.44 (95% CI: 21.05 to 33.82)], with moderate heterogeneity observed (I^2^ = 52.4%, *P *= 0.0074; Supplementary Fig. S3D and E, available at *Rheumatology Advances in Practice* online). Additionally, its ACR70 response rate was significantly higher than that of adalimumab^40mg^ [RD: 11.09 (95% CI: 3.55 to 18.62); Supplementary Fig. S3F, available at *Rheumatology Advances in Practice* online].

## Discussion

Although enthesitis underlies the musculoskeletal manifestations of PsA [3], few studies focus on this domain. Current treatment recommendations present multiple classes of molecular-targeted drugs that are considered effective for treating enthesitis without any preference for therapeutic efficacy. Additionally, no criteria for selecting a drug class in actual clinical practice and even less criteria for selecting individual drugs within a drug class are available. Herein, we conducted an NMA to evaluate treatments for enthesitis according to resolution rates and LEI score reductions. Only RCTs were included, enhancing the reliability of the findings. Results showed that JAKis, especially upadacitinib, demonstrated superior efficacy in treating enthesitis in patients with PsA. At 12 weeks, upadacitinib showed significantly higher enthesitis resolution rates and LEI score reductions than the placebo. These outcomes were significantly enhanced at 24 weeks compared with those under adalimumab. Notably, upadacitinib was the only therapy to demonstrate superior efficacy to adalimumab in resolution rates and LEI score reductions.

Variations in JAKi efficacy for enthesitis may be attributable to differences in JAK isoform selectivity. At 12 weeks, filgotinib did not significantly improve resolution rates compared with the placebo. Similarly, while tofacitinib reduced LEI scores at 12 weeks, this effect was not maintained at 24 weeks. These discrepancies could stem from variations in JAK isoform selectivity. Pathophysiologically, IL-23 and IL-12 are pivotal in PsA development, signalling via the JAK2–tyrosine kinase (TYK) 2 pathway [56]. IL-23 activates Th17 cells to produce IL-17A [10, 57, 58], whereas IL-12 promotes TNF-α production with interferon-γ [59]. Additionally, CD4+ and CD8+ T cells at entheses produce IL-17 and TNF-α, contributing to enthesitis [60]. Thus, inhibiting the JAK2–TYK2 pathway may offer a key therapeutic target for PsA, including enthesitis. At clinical doses, upadacitinib, a JAK1-selective inhibitor, indirectly modulates JAK2–TYK2 cytokines, such as IL-23 and GM-CSF [61–63]. *In vitro* studies assessing JAKis’ effects on JAK2-specific cytokine signalling pathways, particularly GM-CSF/pSTAT3 signalling in monocytes, reported varying inhibition rates: filgotinib^200mg^, 6%; tofacitinib^5mg^, 8%; and upadacitinib 10 mg, upadacitinib^15mg^, and upadacitinib^30mg^, 17%, 27% and 42%, respectively. As observed, upadacitinib was the only JAKi to achieve >50% inhibition over 24 h [61]. Therefore, upadacitinib may more effectively suppress JAK2 cytokines than other JAKis; this result could explain its superior and sustained efficacy against enthesitis in PsA.

LEI score reductions were greater with certolizumab than with adalimumab at 24 weeks. This finding could be attributed to certolizumab’s molecular weight (90.8 kDa, PEGylated) being lower than adalimumab (148 kDa), potentially enhancing its distribution to inflammatory sites [64, 65]. Notably, ozoralizumab, an even smaller TNFi (38.4 kDa), is used to treat rheumatoid arthritis [66] and shows superior distribution to inflammatory sites, with higher concentrations in joint tissues such as the synovium, bone marrow and cartilage (relative to adalimumab) in murine studies [67]. Although not yet tested in PsA, ozoralizumab may be a promising treatment option for enthesitis, especially in poorly vascularized tissues, such as entheses.

Previous meta-analyses have focused primarily on enthesitis resolution rates, often neglecting the importance of LEI score reductions [68–70]. Resolution rates represent the proportion of patients achieving an LEI score of 0, but they do not fully capture the extent of improvement, especially in those with high LEI scores at baseline. Conversely, LEI score reductions provide an absolute measure of treatment efficacy, offering a more nuanced assessment of therapeutic effects. In our study, resolution rates and LEI score reductions were significantly improved with upadacitinib compared with the placebo. Secukinumab also showed significantly improved resolution rates compared with the placebo, but no significant differences were noted in LEI score reductions. Therefore, relying solely on resolution rates may overlook differences in therapeutic efficacy. Hence, both metrics need to be assessed for a more comprehensive evaluation of enthesitis treatments in PsA cases.

We also found significantly higher ACR70 response rates with TNFis, JAKis, IL-17is/IL-17Ris and IL-23is compared with the placebo, consistent with previous studies [71]. Specifically, upadacitinib^30mg^ showed significantly higher rates than adalimumab, demonstrating its effectiveness not only for enthesitis but also joint symptoms in PsA.

However, this study has several limitations. Although low-to-moderate heterogeneity was observed in the drug class–based analyses, additional analyses focusing on individual drugs reduced it; thus, class-based grouping may have obscured differences in treatment effects. Additionally, given the limited data for some therapies, temporal efficacy could not be comprehensively evaluated. Currently, studies focusing on enthesitis in PsA remain scarce, and the findings often rely on small sizes; hence, results should be interpreted cautiously. The safety profile of molecular-targeted therapies in patients with PsA presenting with enthesitis has also not been sufficiently evaluated; thus, we could not conduct an integrated analysis of safety in this specific population. However, safety profiles for these therapies have been investigated by previous meta-analyses focusing on patients with PsA, regardless of enthesitis [70]. Future studies should evaluate the therapies’ safety profiles stratified by enthesitis severity. The current study is also limited by its reliance on LEI scores, which evaluate six sites bilaterally using a binary scale (0/1), thereby limiting its scope. Although LEI scores are widely used in clinical trials, they may not fully capture the extent of systemic enthesitis. Other indices, such as the Spondyloarthritis Research Consortium of Canada and the Maastricht Ankylosing Spondylitis Enthesitis Score, are recognized for being more comprehensive in evaluating systemic enthesitis; however, studies employing these indices were too scarce to include in this analysis [72]. Finally, as the meta-analysis only included studies published up to April 2025, newer therapies were not evaluated. Further clinical trials incorporating emerging treatments are crucial for advancing our understanding of enthesitis management in PsA cases.

## Conclusion

Upadacitinib demonstrated superior efficacy in enthesitis resolution rates and LEI score reductions compared with the placebo and adalimumab groups, while certolizumab showed greater LEI score reductions than adalimumab; therapeutic effects significantly varied even within the same drug class. Thus, the efficacy of individual molecular-targeted therapies for enthesitis in PsA can differ despite belonging to the same drug class. Overall, these findings may inform therapeutic decision-making for enthesitis in PsA. Further direct comparative studies are warranted to validate these results.

## Supplementary Material

rkaf077_Supplementary_Data

## Data Availability

This study protocol has been published in PROSPERO (CRD42024590257). The data used in this study are available in the public domain and can be accessed in the article.
