# Supplementary material for: Therapeutic efficacy of molecular-targeted drugs for enthesitis in patients with PsA: a network meta-analysis
Source: Rheumatol Adv Pract. 2025 Jul 7;9(3):rkaf077. doi: 10.1093/rap/rkaf077 (PMC12270261; doi:10.1093/rap/rkaf077)
Supplement: rkaf077_Supplementary_Data [file rkaf077_supplementary_data.zip › 25-051 Supplementary Material.docx]

**Appendix**

**2**

**16**

**18**

**20**

**23**

**25**

**27**

**29**

**Supplementary Table S1.** **PRISMA-NMA extension checklist**

**Supplementary Table S2.** **Details of the systematic search**

**Supplementary Table S3. Baseline data of studies included in the NMA**

**Supplementary Table S4. The Quality Assessment Tool for Observational Cohort and Cross-Sectional Studies from National Heart, Lung, and Blood Institute (NHLBI)**

**Supplementary Figure S1. Funnel plots evaluating the effect of molecular-targeted**

**drugs on enthesitis resolution and LEI score changes in patients with PsA**

**Supplementary Figure S2. Funnel plot evaluating the effect of molecular-targeted**

**drugs on ACR70 response rates in patients with PsA**

**Supplementary Figure S3. Network diagrams and forest plots evaluating the effect**

**of molecular-targeted drugs on ACR70 response rates in patients with PsA**

**References**

**Supplementary Table S1. PRISMA-NMA extension checklist**

| **Section/Topic** | **Item #** | **Checklist Item** | **Reported in section** |
| --- | --- | --- | --- |
| **TITLE** |  |  |  |
| Title | 1 | Identify the report as a systematic review incorporating a network meta-analysis (or related form of meta-analysis). | Title |
|  |  |  |  |
| **ABSTRACT** |  |  |  |
| Structured summary | 2 | Provide a structured summary including, as applicable:  **Background:** main objectives  **Methods:** data sources; study eligibility criteria, participants, and interventions; study appraisal; and synthesis methods, such as network meta-analysis.  **Results:** number of studies and participants identified; summary estimates with corresponding confidence/credible intervals; treatment rankings may also be discussed. Authors may choose to summarize pairwise comparisons against a chosen treatment included in their analyses for brevity.  **Discussion/Conclusions:** limitations; conclusions and implications of findings.  **Other:** primary source of funding; systematic review registration number with registry name. | Abstract |
|  |  |  |  |
| **INTRODUCTION** |  |  |  |
| Rationale | 3 | Describe the rationale for the review in the context of what is already known, including mention of why a network meta-analysis has been conducted. | Introduction |
| Objectives | 4 | Provide an explicit statement of questions being addressed, with reference to participants, interventions, comparisons, outcomes, and study design (PICOS). | Introduction, Results |
|  |  |  |  |
| **METHODS** |  |  |  |
| Protocol and registration | 5 | Indicate whether a review protocol exists and if and where it can be accessed (e.g., Web address); and, if available, provide registration information, including registration number. | Methods, Table S2 |
| Eligibility criteria | 6 | Specify study characteristics (e.g., PICOS, length of follow-up) and report characteristics (e.g., years considered, language, publication status) used as criteria for eligibility, giving rationale. Clearly describe eligible treatments included in the treatment network, and note whether any have been clustered or merged into the same node (with justification). | Methods, Table S2 |
| Information sources | 7 | Describe all information sources (e.g., databases with dates of coverage, contact with study authors to identify additional studies) in the search and date last searched. | Methods, Table S2 |
| Search | 8 | Present full electronic search strategy for at least one database, including any limits used, such that it could be repeated. | Methods, Table S2 |
| Study selection | 9 | State the process for selecting studies (i.e., screening, eligibility, included in systematic review, and, if applicable, included in the meta-analysis). | Methods, Table S2 |
| Data collection process | 10 | Describe method of data extraction from reports (e.g., piloted forms, independently, in duplicate) and any processes for obtaining and confirming data from investigators. | Methods, Table S2 |
| Data items | 11 | List and define all variables for which data were sought (e.g., PICOS, funding sources) and any assumptions and simplifications made. | Methods, Table S2 |
| Geometry of the network | S1 | Describe methods used to explore the geometry of the treatment network under study and potential biases related to it. This should include how the evidence base has been graphically summarized for presentation, and what characteristics were compiled and used to describe the evidence base to readers. | Methods |
| Risk of bias within individual studies | 12 | Describe methods used for assessing risk of bias of individual studies (including specification of whether this was done at the study or outcome level), and how this information is to be used in any data synthesis. | Methods |
| Summary measures | 13 | State the principal summary measures (e.g., risk ratio, difference in means). Also describe the use of additional summary measures assessed, such as treatment rankings and surface under the cumulative ranking curve (SUCRA) values, as well as modified approaches used to present summary findings from meta-analyses. | Methods |
| Planned methods of analysis | 14 | Describe the methods of handling data and combining results of studies for each network meta-analysis. This should include, but not be limited to:   - Handling of multi-arm trials; - Selection of variance structure; - Selection of prior distributions in Bayesian analyses; and - Assessment of model fit. | Methods |
| Assessment of Inconsistency | S2 | Describe the statistical methods used to evaluate the agreement of direct and indirect evidence in the treatment network(s) studied. Describe efforts taken to address its presence when found. | Methods |
| Risk of bias across studies | 15 | Specify any assessment of risk of bias that may affect the cumulative evidence (e.g., publication bias, selective reporting within studies). | Methods |
| Additional analyses | 16 | Describe methods of additional analyses if done, indicating which were pre-specified. This may include, but not be limited to, the following:   - Sensitivity or subgroup analyses; - Meta-regression analyses; - Alternative formulations of the treatment network; and - Use of alternative prior distributions for Bayesian analyses (if applicable). | Methods |
|  |  |  |  |
| **RESULTS** |  |  |  |
| Study selection | 17 | Give numbers of studies screened, assessed for eligibility, and included in the review, with reasons for exclusions at each stage, ideally with a flow diagram. | Results, Figure 1 |
| Presentation of network structure | S3 | Provide a network graph of the included studies to enable visualization of the geometry of the treatment network. | Results,  Figure 2-5,  Figure S3 |
| Summary of network geometry | S4 | Provide a brief overview of characteristics of the treatment network. This may include commentary on the abundance of trials and randomized patients for the different interventions and pairwise comparisons in the network, gaps of evidence in the treatment network, and potential biases reflected by the network structure. | Results,  Table 1,  Table S3 |
| Study characteristics | 18 | For each study, present characteristics for which data were extracted (e.g., study size, PICOS, follow-up period) and provide the citations. | Results,  Table 1,  Table S3 |
| Risk of bias within studies | 19 | Present data on risk of bias of each study and, if available, any outcome level assessment. | Results,  Table S4 |
| Results of individual studies | 20 | For all outcomes considered (benefits or harms), present, for each study: 1) simple summary data for each intervention group, and 2) effect estimates and confidence intervals. Modified approaches may be needed to deal with information from larger networks. | Results,  Figure 2-5,  Figure S3 |
| Synthesis of results | 21 | Present results of each meta-analysis done, including confidence/credible intervals. In larger networks, authors may focus on comparisons versus a particular comparator (e.g. placebo or standard care), with full findings presented in an appendix. League tables and forest plots may be considered to summarize pairwise comparisons. If additional summary measures were explored (such as treatment rankings), these should also be presented. | Results,  Figure 2-5,  Figure S3 |
| Exploration for inconsistency | S5 | Describe results from investigations of inconsistency. This may include such information as measures of model fit to compare consistency and inconsistency models, *P* values from statistical tests, or summary of inconsistency estimates from different parts of the treatment network. | Results,  Figure 2-5,  Figure S3 |
| Risk of bias across studies | 22 | Present results of any assessment of risk of bias across studies for the evidence base being studied. | Results,  Figure S1, S2,  Table S4 |
| Results of additional analyses | 23 | Give results of additional analyses, if done (e.g., sensitivity or subgroup analyses, meta-regression analyses, alternative network geometries studied, alternative choice of prior distributions for Bayesian analyses, and so forth). | Results,  Figure 3, 5,  Figure S3 |
|  |  |  |  |
| **DISCUSSION** |  |  |  |
| Summary of evidence | 24 | Summarize the main findings, including the strength of evidence for each main outcome; consider their relevance to key groups (e.g., healthcare providers, users, and policy-makers). | Discussion |
| Limitations | 25 | Discuss limitations at study and outcome level (e.g., risk of bias), and at review level (e.g., incomplete retrieval of identified research, reporting bias). Comment on the validity of the assumptions, such as transitivity and consistency. Comment on any concerns regarding network geometry (e.g., avoidance of certain comparisons). | Discussion |
| Conclusions | 26 | Provide a general interpretation of the results in the context of other evidence, and implications for future research. | Discussion |
|  |  |  |  |
| **FUNDING** |  |  |  |
| Funding | 27 | Describe sources of funding for the systematic review and other support (e.g., supply of data); role of funders for the systematic review. This should also include information regarding whether funding has been received from manufacturers of treatments in the network and/or whether some of the authors are content experts with professional conflicts of interest that could affect use of treatments in the network. | Acknowledgements, Contributors, Funding, Conflict of interest statement, Data availability |

PICOS, population, intervention, comparators, outcomes, study design; NMA, network meta-analysis.

**Supplementary Table S2.** **Details of the systematic search**

| **Date of search: January 1986 and April 2025** | |
| --- | --- |
| **Databases** | |
|  | Pubmed (958),  Web of Science (1084),  Scopus (3283)  ClinicalTrials.gov (437) |
| **Search key** | |
|  | “Psoriatic Arthritis” OR “Psoriatic disease” OR “Spondyloarthropathy” OR “Spondyloarthritis” AND “Enthesitis” AND (“Treatment” OR “Therapy”) |
| **Search key expansions** | |
| **Pubmed** | |
|  | “Psoriatic Arthritis” OR “Psoriatic disease” OR “Spondyloarthropathy” OR “Spondyloarthritis” AND “Enthesitis” AND (“Treatment” OR “Therapy”) |
| **Web of Science** | |
|  | ALL= (“Enthesitis” AND (“Treatment” OR “Therapy”) AND (“Psoriatic Arthritis” OR “Psoriatic disease” OR “Spondyloarthropathy” OR “Spondyloarthritis”)) |
| **Scopus** | |
|  | TITLE-ABS-KEY (“Psoriatic Arthritis” OR “Psoriatic disease” OR “Spondyloarthropathy” OR “Spondyloarthritis”) AND ALL (“Enthesitis”) AND TITLE-ABS-KEY (“Treatment” OR “Therapy”) |
| **ClinicalTrials.gov** | |
|  | Other terms: AREA[ConditionSearch](“Psoriatic Arthritis” OR “Psoriatic disease” OR “Spondyloarthropathy” OR “Spondyloarthritis”) AND AREA[BasicSearch](Enthesitis) |

**Supplementary Table S3. Baseline data of studies included in the NMA**

* mean (range).

** medians (interquartile ranges).

LEI, Leeds enthesitis index; MTX, methotrexate; ND, no data; NMA, Network meta-analysis; Q2W, once every 2 weeks; Q4W, once every 4 weeks; Q8W, once every 8 weeks; Q12W, once every 12 weeks; SD, standard deviation.

**Supplementary Table S4. The Quality Assessment Tool for Observational Cohort and Cross-Sectional Studies from National Heart, Lung, and Blood Institute (NHLBI)**

Criteria_1: Was the research question or objective in this paper clearly stated?

Criteria 2: Was the study population clearly specified and defined?

Criteria 3: Was the participation rate of eligible persons at least 50%?

Criteria 4: Were all the subjects selected or recruited from the same or similar populations (including the same time period)? Were inclusion and exclusion criteria for being in the study prespecified and applied uniformly to all participants?

Criteria 5: Was a sample size justification, power description, or variance and effect estimates provided?

Criteria 6: For the analyses in this paper, were the exposure(s) of interest measured prior to the outcome(s) being measured?

Criteria 7: Was the timeframe sufficient so that one could reasonably expect to see an association between exposure and outcome if it existed?

Criteria 8: For exposures that can vary in amount or level, did the study examine different levels of the exposure as related to the outcome (e.g., categories of exposure, or exposure measured as continuous variable)?

Criteria 9: Were the exposure measures (independent variables) clearly defined, valid, reliable, and implemented consistently across all study participants?

Criteria 10: Was the exposure(s) assessed more than once over time?

Criteria 11: Were the outcome measures (dependent variables) clearly defined, valid, reliable, and implemented consistently across all study participants?

Criteria 12: Were the outcome assessors blinded to the exposure status of participants?

Criteria 13: Was loss to follow-up after baseline 20% or less?

Criteria 14: Were key potential confounding variables measured and adjusted statistically for their impact on the relationship between exposure(s) and outcome(s)?

N/A, not available.

**Supplementary Figure S1. Funnel plots evaluating the effect of molecular-targeted drugs on enthesitis resolution and LEI score changes in patients with psoriatic arthritis**

(A, B) Funnel plots show the effect of individual molecular-targeted drugs on enthesitis resolution at 12 weeks (A) and 24 weeks (B) of administration in patients with psoriatic arthritis (PsA). (C, D) Funnel plots show the effect of individual molecular-targeted drugs on LEI score changes at 12 weeks (C) and 24 weeks (D) of administration in patients with PsA.

ABT, abatacept; ADA, adalimumab; BKZ, bimekizumab; BRO, brodalumab; CEZ, certolizumab pegol; FIL, filgotinib; GUS, guselkumab; IXE, ixekizumab; LEI, Leeds enthesitis index; plac, placebo; Q2W, once every 2 weeks; Q4W, once every 4 weeks; Q8W, once every 8 weeks; Q12W, once every 12 weeks; RZB, risankizumab; SEC, secukinumab; TIL, tildrakizumab; TOF, tofacitinib; UPA, upadacitinib.

**Supplementary Figure S2. Funnel plot evaluating the effect of molecular-targeted drugs on ACR70 response rates in PsA patients with psoriatic arthritis**

Funnel plot shows the effect of individual molecular-targeted drugs on ACR70 response rates at 12–24 weeks in patients with psoriatic arthritis (PsA).

ABT, abatacept; ACR, American College of Rheumatology; ADA, adalimumab; BKZ, bimekizumab; BRO, brodalumab; CEZ, certolizumab pegol; FIL, filgotinib; GUS, guselkumab; IXE, ixekizumab; plac, placebo; Q2W, once every 2 weeks; Q4W, once every 4 weeks; Q8W, once every 8 weeks; Q12W, once every 12 weeks; RZB, risankizumab; SEC, secukinumab; TIL, tildrakizumab; TOF, tofacitinib; UPA, upadacitinib.

**Supplementary Figure S3. Network diagrams and forest plots evaluating the effect of molecular-targeted drugs on ACR70 response rates in patients with psoriatic arthritis**

Drug class–based evaluation (A, B, C): (A) Network diagram shows the ACR70 response rates at 12–24 weeks of administering various molecular-targeted drugs to patients with psoriatic arthritis (PsA). The node size is proportional to the total number of patients randomized to each treatment; the edge line thickness is proportional to the total number of studies informing each comparison. (B) Forest plot presents the ACR70 response rates of such drugs compared with those of placebo, evaluated using risk differences with 95% confidence intervals, which were estimated using a random-effects model. (C) Forest plot present the ACR70 response rates of such drugs compared with those of TNFi, evaluated using risk differences. Evaluation of individual molecular-targeted drugs (D, E, F): (D) Network diagram shows the ACR70 response rates at 12–24 weeks of administering individual molecular-targeted drugs to patients with PsA. The node size is proportional to the total number of patients randomized to each treatment; the edge line thickness is proportional to the total number of studies informing each comparison. (E) Forest plot presents the ACR70 response rates of such drugs compared with those of placebo, evaluated using risk differences with 95% confidence intervals, which were estimated using a random-effects model. (F) Forest plot presents the ACR70 response rates of such drugs compared with those of adalimumab, evaluated using risk differences. Heterogeneity across studies was assessed using the I² statistic.

CTLA-4Ig, cytotoxic T lymphocyte–associated antigen-4 immunoglobulin; IL-17i, interleukin-17 inhibitor; IL-17Ri, interleukin-17 receptor inhibitor; IL-23i, interleukin-23 inhibitor; JAKi, Janus kinase inhibitor; Q2W, once every 2 weeks; Q4W, once every 4 weeks; Q8W, once every 8 weeks; Q12W, once every 12 weeks; RD, risk difference; TNFi, tumour necrosis factor–alpha inhibitor; 95% CI, 95% confidence intervals.

**References**

1. Mease PJ, Gottlieb AB, Heijde DVD, FitzGerald O, Johnsen A, Nys M, et al. Efficacy and safety of abatacept, a T-cell modulator, in a randomised, double-blind, placebo-controlled, phase III study in psoriatic arthritis. Ann Rheum Dis. 2017;76(9):1550–8.
2. Mease PJ, Merola JF, Tanaka Y, Gossec L, McInnes IB, Ritchlin CT, et al. Safety and Efficacy of Bimekizumab in Patients with Psoriatic Arthritis: 2-Year Results from Two Phase 3 Studies. Rheumatol Ther. 2024;11:1363–1382.
3. Mease PJ, Genovese MC, Greenwald MW, Ritchlin CT, Beaulieu AD, Deodhar A, et al. Brodalumab, an anti-IL17RA monoclonal antibody, in psoriatic arthritis. N Engl J Med. 2014;370(24):2295–306.
4. Mease PJ, Helliwell PS, Hjuler KF, Raymond K, McInnes IB. Brodalumab in psoriatic arthritis: results from the randomized phase III AMVISION-1 and AMVISION-2 trials. Ann Rheum Dis. 2021;80(2):185–93.
5. Mease PJ, Fleischmann R, Deodhar AA, Wollenhaupt J, Khraishi M, Kielar D, et al. Effect of certolizumab pegol on signs and symptoms in patients with psoriatic arthritis: 24-week results of a phase 3 double-blind randomised placebo-controlled study (RAPID-PsA). Ann Rheum Dis. 2014;73(1):48–55.
6. Mease PJ, Coates LC, Helliwell PS, Stanislavchuk M, Rychlewska-Hanczewska A, Dudek A, et al. Efficacy and safety of filgotinib, a selective Janus kinase 1 inhibitor, in patients with active psoriatic arthritis (EQUATOR): results from a randomized, placebo-controlled, phase 2 trial. Lancet. 2018;392(10162):2367–77.
7. Mease PJ, Gladman DD, Deodhar A, McGonagle DG, Nash P, Boehncke WH, et al. Impact of guselkumab, an interleukin-23 p19 subunit inhibitor, on enthesitis and dactylitis in patients with moderate to severe psoriatic arthritis: results from a randomized, placebo-controlled, phase II study. RMD Open. 2020;6(2):e001217.
8. Deodhar A, Helliwell PS, Boehncke WH, Kollmeier AP, Hsia EC, Subramanian RA, et al. Guselkumab in patients with active psoriatic arthritis who were biologic-naive or had previously received TNFα inhibitor treatment (DISCOVER-1): a double-blind, randomised, placebo-controlled phase 3 trial. Lancet. 2020;395(10230):1115–1125.
9. McInnes IB, Rahman P, Gottlieb AB, Hsia EC, Kollmeier AP, Xu XL, et al. Long-term efficacy and safety of guselkumab, a monoclonal antibody specific to the p19 subunit of interleukin-23, through two years: results from a phase III, randomized, double-blind, placebo-controlled study conducted in biologic-naive patients with active psoriatic arthritis. Arthritis Rheumatol. 2022;74(3):475–85.
10. McGonagle D, McInnes IB, Deodhar A, Schett G, Shawi M, Kafka S, et al. Resolution of enthesitis by guselkumab and relationships to disease burden: 1-year results of two phase 3 psoriatic arthritis studies. Rheumatology. 2021;60(11):5337–50.
11. Mease PJ, Heijde DVD, Ritchlin CT, Okada M, Cuchacovich RS, Shuler CL, et al. Ixekizumab, an interleukin-17A specific monoclonal antibody, for the treatment of biologic-naive patients with active psoriatic arthritis: results from the 24-week randomised, double-blind, placebo-controlled and active (adalimumab)-controlled period of the phase III trial SPIRIT-P1. Ann Rheum Dis. 2017;76(1):79–87.
12. Nash P, Kirkham B, Okada M, Rahman P, Combe B, Burmester GR, et al. Ixekizumab for the treatment of patients with active psoriatic arthritis and an inadequate response to tumour necrosis factor inhibitors: results from the 24-week randomised, double-blind, placebo-controlled period of the SPIRIT-P2 phase 3 trial. Lancet. 2017;389(10086):2317–27.
13. Gladman DD, Orbai AM, Klitz U, Wei JCC, Gallo G, Birt J, et al. Ixekizumab and complete resolution of enthesitis and dactylitis: integrated analysis of two phase 3 randomized trials in psoriatic arthritis. Arthritis Res Ther. 2019;21(1):38.
14. Kristensen LE, Okada M, Tillett W, Leage SL, Baou CE, Sapin C, et al. Ixekizumab Demonstrates Consistent Efficacy Versus Adalimumab in Biologic Disease-Modifying Anti-rheumatic Drug-Naïve Psoriatic Arthritis Patients Regardless of Psoriasis Severity: 52-Week Post Hoc Results from SPIRIT-H2H. Rheumatol Ther. 2022;9(1):109–125.
15. Östör A, Bosch FVD, Papp K, Asnal C, Blanco R, Aelion J, et al. Efficacy and safety of risankizumab for active psoriatic arthritis: 24-week results from the randomized, double-blind, phase 3 KEEPsAKE 2 trial. Ann Rheum Dis. 2022;81(3):351–8.
16. Kwatra SG, Khattri S, Amin AZ, Ranza R, Kaplan B, Shi L, et al. Enthesitis and dactylitis resolution with risankizumab for active psoriatic arthritis: integrated analysis of the randomized KEEPsAKE 1 and 2 trials. Dermatol Ther (Heidelb). 2024;14(6):1517–30.
17. Nash P, Mease PJ, McInnes IB, Rahman P, Ritchlin CT, Blanco R, et al. Efficacy and safety of secukinumab administration by autoinjector in patients with psoriatic arthritis: results from a randomized, placebo-controlled trial (FUTURE 3). Arthritis Res Ther. 2018;20(1):47.
18. Mease PJ, Heijde DVD, Landewé R, Mpofu S, Rahman P, Tahir H, et al. Secukinumab improves active psoriatic arthritis symptoms and inhibits radiographic progression: primary results from the randomised, double-blind, phase III FUTURE 5 study. Ann Rheum Dis. 2018;77(6):890–897.
19. McInnes IB, Behrens F, Mease PJ, Kavanaugh A, Ritchlin C, Nash P, et al. Secukinumab versus adalimumab for treatment of active psoriatic arthritis (EXCEED): a double-blind, parallel-group, randomised, active-controlled, phase 3b trial. Lancet. 2020;395(10235):1496–1505.
20. Kaeley GS, Schett G, Conaghan PG, McGonagle D, Behrens F, Goupille P, et al. Enthesitis in patients with psoriatic arthritis treated with secukinumab or adalimumab: a post hoc analysis of the EXCEED study. Rheumatology. 2024;63(1):41–9.
21. Mease PJ, Chohan S, Fructuoso FJG, Luggen ME, Rahman P, Raychaudhuri SP, et al. Efficacy and safety of tildrakizumab in patients with active psoriatic arthritis: results of a randomised, double-blind, placebo-controlled, multiple-dose, 52-week phase IIb study. Ann Rheum Dis. 2021;80(9):1147–1157.
22. Mease P, Hall S, FitzGerald O, Heijde DVD, Merola JF, Avila-Zapata F, et al. Tofacitinib or adalimumab versus placebo for psoriatic arthritis. N Engl J Med. 2017;377(16):1537–50.
23. Gladman D, Rigby W, Azevedo VF, Behrens F, Blanco R, Kaszuba A, et al. Tofacitinib for psoriatic arthritis in patients with an inadequate response to TNF inhibitors. N Engl J Med. 2017;377(16):1525–36.
24. Nash P, Coates LC, Fleischmann R, Papp KA, Gomez-Reino JJ, Kanik KS, et al. Efficacy of Tofacitinib for the Treatment of Psoriatic Arthritis: Pooled Analysis of Two Phase 3 Studies. Rheumatol Ther. 2018;5(2):567–582.
25. McInnes IB, Kato K, MarMagrey M, Merola JF, Kishimoto M, Haaland D, et al. Efficacy and Safety of Upadacitinib in Patients with Psoriatic Arthritis: 2-Year Results from the Phase 3 SELECT-PsA 1 Study. Rheumatol Ther. 2023;10(1):275–292.
26. Mease PJ, Lertratanakul A, Anderson JK, Papp K, Bosch FVD, Tsuji S, et al. Upadacitinib for psoriatic arthritis refractory to biologics: SELECT-PsA 2. Ann Rheum Dis. 2021;80(3):312–20.
27. Cantini F, Marchesoni A, Novelli L, Gualberti G, Marando F, McDearmon-Blondell EL, et al. Effects of upadacitinib on enthesitis in patients with psoriatic arthritis: a post hoc analysis of SELECT-PsA 1 and 2 trials. Rheumatology. 2024;63(11):3146–54.
28. Araujo EG, Englbrecht M, Hoepken S, Finzel S, Kampylafka E, Kleyer A, et al. Effects of ustekinumab versus tumor necrosis factor inhibition on enthesitis: Results from the enthesial clearance in psoriatic arthritis (ECLIPSA) study. Semin Arthritis Rheum. 2019;48(4):632–637.
